# Chlorido(4,4′,4′′-tri-*tert*-butyl-2,2′:6′,2′′-terpyridine)­platinum(II) tetra­fluorido­borate

**DOI:** 10.1107/S1600536810048762

**Published:** 2010-11-30

**Authors:** Rami J. Batrice, Vladimir N. Nesterov, Bradley W. Smucker

**Affiliations:** a Department of Chemistry, Austin College, 900 North Grand, Sherman, TX 75090-4400, USA; b Department of Chemistry, University of North Texas, 1155 Union Circle, #305070, Denton, TX 76203-5070, USA

## Abstract

In the title compound, [PtCl(C_27_H_35_N_3_)]BF_4_, the Pt^II^ atom is in a pseudo-square-planar coordination, which is typical of Pt–terpyridine complexes. The Pt—Cl bond distance is 2.2998 (7) Å. The Pt—N distance of the N atom on the central pyridine is 1.931 (2) Å, while the peripheral N atoms have Pt—N distances of 2.018 (2) and 2.022 (2) Å. The cations pack as dimers in a head-to-tail orientation with an inter­molecular Pt⋯Pt distance of 3.5214 (2) Å and Pt⋯N distances of 3.527 (2), 3.873 (2) and 4.532 (2) Å. In the crystal, cations and anions are linked by weak C—H⋯F hydrogen-bonding inter­actions.

## Related literature

For other crystal structures of the title cation, [(tbtrpy)PtCl]^+^, see: Batrice *et al.* (2010 ▶); Lai *et al.* (1999 ▶). For related terpyridine complexes with close inter­molecular Pt⋯Pt distances, see: Angle *et al.* (2006 ▶); Bailey *et al.* (1995 ▶). For synthetic procedures, see: Howe-Grant & Lippard (1980 ▶).
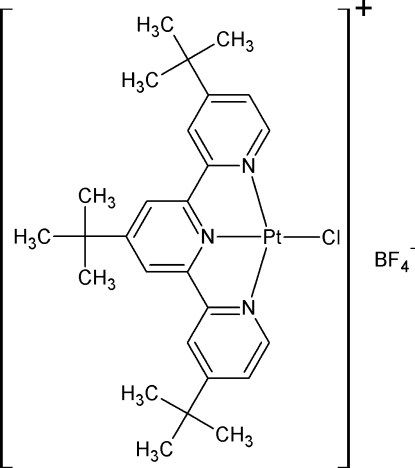


## Experimental

### 

#### Crystal data

[PtCl(C_27_H_35_N_3_)]BF_4_
*M* *_r_* = 718.93
Monoclinic, 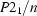

*a* = 12.5921 (7) Å
*b* = 16.4998 (9) Å
*c* = 13.3262 (7) Å
β = 92.239 (1)°
*V* = 2766.6 (3) Å^3^
*Z* = 4
Mo *K*α radiation
μ = 5.22 mm^−1^
*T* = 100 K
0.35 × 0.12 × 0.09 mm

#### Data collection

Bruker SMART APEXII CCD diffractometer
Absorption correction: numerical (*SADABS*; Bruker, 2008 ▶) *T*_min_ = 0.266, *T*_max_ = 0.657
24815 measured reflections
6116 independent reflections
5415 reflections with *I* > 2σ(*I*)
*R*_int_ = 0.027

#### Refinement

*R*[*F* ^2^ > 2σ(*F* ^2^)] = 0.020
*wR*(*F* ^2^) = 0.058
*S* = 1.00
6116 reflections
343 parameters
H-atom parameters constrained
Δρ_max_ = 1.93 e Å^−3^
Δρ_min_ = −0.89 e Å^−3^

### 

Data collection: *APEX2* (Bruker, 2007 ▶); cell refinement: *SAINT* (Bruker, 2007 ▶); data reduction: *SAINT*; program(s) used to solve structure: *SHELXS97* (Sheldrick, 2008 ▶); program(s) used to refine structure: *SHELXL97* (Sheldrick, 2008 ▶); molecular graphics: *SHELXTL* (Sheldrick, 2008 ▶); software used to prepare material for publication: *SHELXTL* and *Mercury* (Macrae *et al.*, 2008 ▶).

## Supplementary Material

Crystal structure: contains datablocks global, I. DOI: 10.1107/S1600536810048762/pv2335sup1.cif

Structure factors: contains datablocks I. DOI: 10.1107/S1600536810048762/pv2335Isup2.hkl

Additional supplementary materials:  crystallographic information; 3D view; checkCIF report

## Figures and Tables

**Table 1:** Hydrogen-bond geometry (Å, °)

*D*—H⋯*A*	*D*—H	H⋯*A*	*D*⋯*A*	*D*—H⋯*A*
C1—H1*A*⋯F3^i^	0.95	2.51	3.330 (3)	145
C2—H2*A*⋯F1^i^	0.95	2.36	3.229 (3)	151
C7—H7*A*⋯F2^ii^	0.95	2.46	3.333 (3)	154
C17—H17*B*⋯F4^ii^	0.98	2.36	3.295 (3)	159
C27—H27*C*⋯F3^iii^	0.98	2.48	3.349 (4)	147
C9—H9*A*⋯F4	0.95	2.39	3.250 (3)	150
C12—H12*A*⋯F4	0.95	2.49	3.298 (3)	142

Symmetry codes: (i) 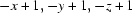; (ii) 
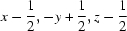
; (iii) 
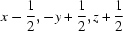
.

**Fig. 1.**
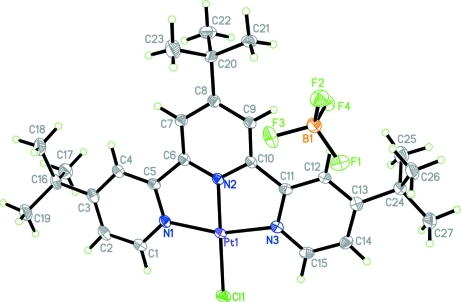
View of title complex (50% probability displacement ellipsoids)

**Fig. 2.**
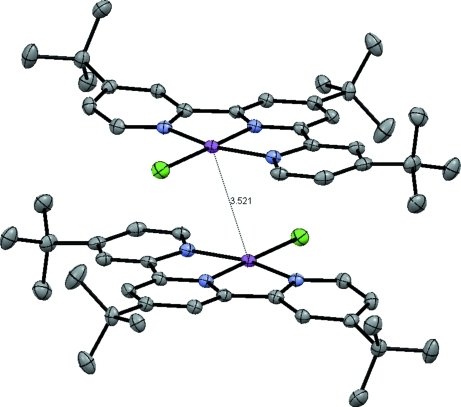
Mercury (Macrae et al., 2008) rendition of head-to-tail packing with Pt—Pt' distance 3.5214 (2) Å. Symmetry operation for the primed atom: 1-x, 1-y, 1-z

## supplementary crystallographic information

### Comment

In the structures of the [(tbtrpy)PtCl]^+^ (tbtrpy = 4,4',4''-tri- *tert*-butyl-2,2':6',2''-terpyridine) complexes with either chloride (Batrice *et al.*, 2010), tetrafluoroborate (title complex) or perchlorate (Lai *et al.*, 1999) counterions, the bond distances and angles around the platinum atom are all similar. The cations in these structures all pack as dimers in a head-to-tail orientation. Interestingly, the interplanar (Pt, Cl and N atoms) distance seems to be related to the size of the anion with the Cl^-^, BF_4_^-^, and ClO_4_^-^ being 3.283, 3.390 and 3.536 Å, respectively. In addition to the size of the counterion, solvent is also noted as playing a role in the ability of these types of complexes to interact significantly with each other (Bailey  *et al.*, 1995). In the title complex, the cations and anions are linked by weak H-bonding interactions between C—H···F (Table 1).

The intermolecular distance is within a suitable distance for favorable π-π interactions. So the bulky *tert*-Butyl groups of the tbtrpy ligand do not appear to alter the ability of this complex to form suitable interactions between the two molecules of the dimer. The difference in color between the crystal (red) and the powder (yellow) is, likewise, attributed to this dimer interaction (Angle  *et al.*, 2006).

### Experimental

[(tbtrpy)PtCl]Cl was synthesized according to modifications on a published procedure (Howe-Grant and Lippard, 1980). This [(tbtrpy)PtCl]^+^ complex was reacted with various aromatic thiol ligands (SAr). One such product containing a [(tbtrpy)Pt(SAr)]Cl (0.02 mmol) complex was reacted with sodium tetrafluoroborate (0.10 mmol) in a solution of methanol and isolated through condensation and precipitation with diethyl ether (94% yield). Crystals of the title compound were grown from the vapor diffusion of cyclohexane into a dichloromethane solution containing [(tbtrpy)Pt(SAr)]BF_4_ and [(tbtrpy)PtCl]BF_4_.

### Refinement

H atoms were placed in idealized positions with C—H = 0.95 and 0.98 Å for aryl and methyl H-atoms, respectively, and allowed to ride on their parent atoms with *U*_iso_(H) 1.2 (aryl C) or 1.5 (methyl C) × *U*_eq_ of the parent atoms. The largest residual electron density in the final difference map was located close to the platinum atom (0.83 Å) and was most likely due to imperfect absorption corrections frequently encountered in heavy-metal atom structures.

### Crystal data

**Table:**

[PtCl(C_27_H_35_N_3_)]BF_4_	*F*(000) = 1416
*M**_r_* = 718.93	*D*_x_ = 1.726 Mg m^−^^3^
Monoclinic, *P*2_1_/*n*	Mo *K*α radiation, λ = 0.71073 Å
Hall symbol: -P 2yn	Cell parameters from 9942 reflections
*a* = 12.5921 (7) Å	θ = 2.2–27.1°
*b* = 16.4998 (9) Å	µ = 5.22 mm^−^^1^
*c* = 13.3262 (7) Å	*T* = 100 K
β = 92.239 (1)°	Plate, red
*V* = 2766.6 (3) Å^3^	0.35 × 0.12 × 0.09 mm
*Z* = 4	

### Data collection

**Table:**

Bruker SMART APEXII CCD diffractometer	6116 independent reflections
Radiation source: fine-focus sealed tube	5415 reflections with *I* > 2σ(*I*)
graphite	*R*_int_ = 0.027
ω scans	θ_max_ = 27.2°, θ_min_ = 2.0°
Absorption correction: numerical (*SADABS*; Bruker, 2008)	*h* = −16→16
*T*_min_ = 0.266, *T*_max_ = 0.657	*k* = −21→21
24815 measured reflections	*l* = −17→17

### Refinement

**Table:**

Refinement on *F*^2^	Primary atom site location: structure-invariant direct methods
Least-squares matrix: full	Secondary atom site location: difference Fourier map
*R*[*F*^2^ > 2σ(*F*^2^)] = 0.020	Hydrogen site location: inferred from neighbouring sites
*wR*(*F*^2^) = 0.058	H-atom parameters constrained
*S* = 1.00	*w* = 1/[σ^2^(*F*_o_^2^) + (0.040*P*)^2^] where *P* = (*F*_o_^2^ + 2*F*_c_^2^)/3
6116 reflections	(Δ/σ)_max_ = 0.004
343 parameters	Δρ_max_ = 1.93 e Å^−^^3^
0 restraints	Δρ_min_ = −0.89 e Å^−^^3^

### Special details

**Table:**

Geometry. All e.s.d.'s (except the e.s.d. in the dihedral angle between two l.s. planes) are estimated using the full covariance matrix. The cell e.s.d.'s are taken into account individually in the estimation of e.s.d.'s in distances, angles and torsion angles; correlations between e.s.d.'s in cell parameters are only used when they are defined by crystal symmetry. An approximate (isotropic) treatment of cell e.s.d.'s is used for estimating e.s.d.'s involving l.s. planes.
Refinement. Refinement of *F*^2^ against ALL reflections. The weighted *R*-factor *wR* and goodness of fit *S* are based on *F*^2^, conventional *R*-factors *R* are based on *F*, with *F* set to zero for negative *F*^2^. The threshold expression of *F*^2^ > σ(*F*^2^) is used only for calculating *R*-factors(gt) *etc*. and is not relevant to the choice of reflections for refinement. *R*-factors based on *F*^2^ are statistically about twice as large as those based on *F*, and *R*- factors based on ALL data will be even larger.

### Fractional atomic coordinates and isotropic or equivalent isotropic displacement parameters (Å^2^)

**Table:**

	*x*	*y*	*z*	*U*_iso_*/*U*_eq_	
Pt1	0.387307 (7)	0.441874 (6)	0.526749 (7)	0.01521 (5)	
Cl1	0.31903 (6)	0.53400 (4)	0.63655 (5)	0.02397 (15)	
N1	0.35302 (17)	0.49919 (13)	0.39565 (16)	0.0171 (5)	
N2	0.44357 (17)	0.36376 (13)	0.43485 (16)	0.0152 (4)	
N3	0.43642 (17)	0.35687 (13)	0.62754 (16)	0.0168 (4)	
C1	0.3043 (2)	0.57117 (15)	0.3825 (2)	0.0199 (6)	
H1A	0.2845	0.6011	0.4397	0.024*	
C2	0.2822 (2)	0.60272 (16)	0.2876 (2)	0.0226 (6)	
H2A	0.2490	0.6542	0.2809	0.027*	
C3	0.3078 (2)	0.56012 (15)	0.2020 (2)	0.0218 (6)	
C4	0.3612 (2)	0.48624 (15)	0.2170 (2)	0.0192 (6)	
H4A	0.3824	0.4558	0.1607	0.023*	
C5	0.3830 (2)	0.45736 (15)	0.3128 (2)	0.0168 (5)	
C6	0.4388 (2)	0.37982 (15)	0.3360 (2)	0.0170 (5)	
C7	0.4844 (2)	0.32631 (16)	0.2701 (2)	0.0185 (5)	
H7A	0.4801	0.3366	0.1999	0.022*	
C8	0.5366 (2)	0.25723 (16)	0.3070 (2)	0.0179 (5)	
C9	0.5381 (2)	0.24221 (16)	0.41048 (19)	0.0167 (5)	
H9A	0.5719	0.1950	0.4372	0.020*	
C10	0.4901 (2)	0.29631 (15)	0.47348 (19)	0.0165 (5)	
C11	0.4815 (2)	0.29055 (16)	0.5836 (2)	0.0170 (5)	
C12	0.5092 (2)	0.22285 (15)	0.6392 (2)	0.0175 (5)	
H12A	0.5418	0.1784	0.6072	0.021*	
C13	0.4903 (2)	0.21825 (16)	0.7415 (2)	0.0192 (5)	
C14	0.4492 (2)	0.28732 (17)	0.7852 (2)	0.0213 (6)	
H14A	0.4377	0.2880	0.8552	0.026*	
C15	0.4246 (2)	0.35546 (16)	0.7275 (2)	0.0197 (6)	
H15A	0.3987	0.4025	0.7596	0.024*	
C16	0.2742 (2)	0.59045 (17)	0.0967 (2)	0.0243 (6)	
C17	0.1605 (3)	0.55930 (17)	0.0725 (3)	0.0314 (7)	
H17A	0.1127	0.5794	0.1231	0.047*	
H17B	0.1604	0.4999	0.0729	0.047*	
H17C	0.1363	0.5788	0.0060	0.047*	
C18	0.3479 (3)	0.55840 (19)	0.0164 (3)	0.0341 (8)	
H18A	0.4210	0.5754	0.0328	0.051*	
H18B	0.3252	0.5803	−0.0494	0.051*	
H18C	0.3443	0.4991	0.0145	0.051*	
C19	0.2725 (3)	0.68348 (17)	0.0921 (2)	0.0321 (7)	
H19A	0.2222	0.7043	0.1402	0.048*	
H19B	0.2504	0.7010	0.0242	0.048*	
H19C	0.3437	0.7045	0.1091	0.048*	
C20	0.5899 (2)	0.19879 (17)	0.2353 (2)	0.0214 (6)	
C21	0.6566 (2)	0.13425 (17)	0.2912 (2)	0.0230 (6)	
H21A	0.7109	0.1607	0.3345	0.035*	
H21B	0.6910	0.0995	0.2426	0.035*	
H21C	0.6104	0.1013	0.3325	0.035*	
C22	0.5025 (3)	0.15485 (19)	0.1712 (2)	0.0322 (7)	
H22A	0.4562	0.1247	0.2154	0.048*	
H22B	0.5356	0.1172	0.1250	0.048*	
H22C	0.4603	0.1948	0.1327	0.048*	
C23	0.6620 (3)	0.2465 (2)	0.1672 (3)	0.0404 (9)	
H23A	0.7130	0.2782	0.2084	0.061*	
H23B	0.6187	0.2831	0.1245	0.061*	
H23C	0.7004	0.2088	0.1249	0.061*	
C24	0.5084 (2)	0.13826 (17)	0.7973 (2)	0.0232 (6)	
C25	0.4381 (3)	0.07374 (18)	0.7441 (3)	0.0329 (7)	
H25A	0.4637	0.0639	0.6767	0.049*	
H25B	0.3645	0.0930	0.7389	0.049*	
H25C	0.4413	0.0233	0.7829	0.049*	
C26	0.6249 (2)	0.11286 (18)	0.7924 (2)	0.0290 (7)	
H26A	0.6437	0.1076	0.7220	0.044*	
H26B	0.6354	0.0607	0.8265	0.044*	
H26C	0.6702	0.1540	0.8255	0.044*	
C27	0.4771 (3)	0.14391 (19)	0.9062 (2)	0.0365 (8)	
H27A	0.5214	0.1846	0.9413	0.055*	
H27B	0.4875	0.0911	0.9388	0.055*	
H27C	0.4022	0.1597	0.9087	0.055*	
F1	0.78839 (14)	0.21514 (10)	0.65679 (12)	0.0296 (4)	
F2	0.88980 (12)	0.16408 (12)	0.53331 (11)	0.0282 (4)	
F3	0.76581 (14)	0.26159 (10)	0.49683 (14)	0.0329 (4)	
F4	0.71285 (13)	0.13398 (9)	0.53646 (12)	0.0254 (4)	
B1	0.7889 (3)	0.19389 (19)	0.5557 (2)	0.0213 (6)	

### Atomic displacement parameters (Å^2^)

**Table:**

	*U*^11^	*U*^22^	*U*^33^	*U*^12^	*U*^13^	*U*^23^
Pt1	0.01385 (7)	0.01259 (6)	0.01913 (7)	0.00101 (4)	0.00005 (4)	−0.00161 (4)
Cl1	0.0266 (4)	0.0180 (3)	0.0276 (4)	0.0041 (3)	0.0058 (3)	−0.0037 (3)
N1	0.0166 (11)	0.0140 (11)	0.0206 (11)	0.0007 (9)	−0.0031 (9)	0.0013 (9)
N2	0.0138 (11)	0.0134 (10)	0.0183 (11)	0.0010 (8)	−0.0015 (9)	−0.0002 (8)
N3	0.0138 (11)	0.0164 (11)	0.0202 (11)	0.0001 (9)	0.0005 (9)	−0.0008 (9)
C1	0.0171 (14)	0.0149 (13)	0.0275 (15)	0.0003 (10)	−0.0011 (11)	−0.0018 (11)
C2	0.0193 (14)	0.0147 (13)	0.0336 (16)	0.0017 (11)	−0.0021 (12)	0.0040 (11)
C3	0.0174 (14)	0.0182 (14)	0.0295 (16)	−0.0041 (10)	−0.0005 (12)	0.0041 (11)
C4	0.0182 (14)	0.0164 (13)	0.0229 (14)	−0.0008 (10)	−0.0005 (11)	0.0010 (11)
C5	0.0144 (13)	0.0136 (12)	0.0222 (14)	−0.0020 (10)	−0.0017 (11)	−0.0029 (10)
C6	0.0153 (13)	0.0158 (12)	0.0195 (13)	0.0011 (10)	−0.0039 (10)	0.0000 (10)
C7	0.0196 (14)	0.0191 (13)	0.0165 (12)	0.0005 (11)	−0.0022 (10)	−0.0015 (11)
C8	0.0169 (13)	0.0169 (13)	0.0197 (13)	0.0010 (10)	−0.0006 (10)	−0.0003 (10)
C9	0.0155 (13)	0.0152 (12)	0.0194 (13)	0.0017 (10)	−0.0009 (10)	−0.0005 (10)
C10	0.0166 (13)	0.0132 (12)	0.0195 (13)	0.0001 (10)	−0.0016 (10)	0.0002 (10)
C11	0.0133 (13)	0.0166 (12)	0.0211 (13)	0.0013 (10)	0.0007 (10)	−0.0028 (10)
C12	0.0183 (14)	0.0152 (12)	0.0192 (13)	0.0013 (10)	0.0012 (10)	−0.0016 (10)
C13	0.0159 (13)	0.0198 (13)	0.0218 (14)	−0.0007 (11)	0.0007 (11)	0.0017 (11)
C14	0.0207 (14)	0.0259 (14)	0.0174 (13)	0.0027 (12)	0.0033 (11)	−0.0012 (11)
C15	0.0163 (13)	0.0206 (13)	0.0223 (14)	0.0023 (11)	0.0047 (11)	−0.0046 (11)
C16	0.0271 (16)	0.0189 (14)	0.0267 (15)	0.0042 (12)	−0.0018 (12)	0.0076 (12)
C17	0.0337 (19)	0.0297 (17)	0.0299 (17)	0.0002 (13)	−0.0086 (14)	0.0031 (13)
C18	0.041 (2)	0.0301 (18)	0.0312 (18)	0.0074 (14)	0.0066 (15)	0.0129 (13)
C19	0.0403 (19)	0.0228 (15)	0.0330 (17)	0.0005 (14)	−0.0016 (14)	0.0083 (13)
C20	0.0250 (15)	0.0217 (14)	0.0177 (13)	0.0079 (11)	0.0037 (11)	0.0003 (11)
C21	0.0238 (15)	0.0229 (14)	0.0222 (14)	0.0061 (12)	0.0002 (11)	−0.0040 (11)
C22	0.0386 (19)	0.0310 (17)	0.0263 (16)	0.0120 (14)	−0.0073 (14)	−0.0090 (13)
C23	0.045 (2)	0.0331 (18)	0.045 (2)	0.0127 (16)	0.0243 (17)	0.0129 (15)
C24	0.0305 (16)	0.0184 (13)	0.0210 (14)	0.0036 (12)	0.0043 (12)	0.0011 (11)
C25	0.038 (2)	0.0274 (16)	0.0330 (18)	−0.0066 (14)	0.0034 (15)	0.0073 (13)
C26	0.0330 (18)	0.0231 (15)	0.0309 (16)	0.0058 (13)	0.0001 (13)	0.0070 (13)
C27	0.056 (2)	0.0277 (16)	0.0263 (16)	0.0092 (15)	0.0129 (15)	0.0065 (13)
F1	0.0309 (10)	0.0321 (10)	0.0259 (9)	0.0006 (8)	0.0025 (7)	−0.0067 (7)
F2	0.0232 (10)	0.0361 (11)	0.0255 (9)	0.0054 (7)	0.0044 (7)	−0.0007 (7)
F3	0.0332 (10)	0.0228 (9)	0.0422 (11)	−0.0023 (7)	−0.0048 (8)	0.0117 (8)
F4	0.0261 (9)	0.0195 (8)	0.0305 (9)	−0.0023 (7)	−0.0012 (7)	0.0009 (7)
B1	0.0222 (16)	0.0194 (16)	0.0223 (16)	0.0004 (12)	0.0002 (13)	0.0005 (12)

### Geometric parameters (Å, °)

**Table:**

Pt1—N2	1.931 (2)	C17—H17A	0.9800
Pt1—N1	2.018 (2)	C17—H17B	0.9800
Pt1—N3	2.022 (2)	C17—H17C	0.9800
Pt1—Cl1	2.2998 (7)	C18—H18A	0.9800
N1—C1	1.345 (3)	C18—H18B	0.9800
N1—C5	1.368 (3)	C18—H18C	0.9800
N2—C6	1.343 (3)	C19—H19A	0.9800
N2—C10	1.350 (3)	C19—H19B	0.9800
N3—C15	1.346 (3)	C19—H19C	0.9800
N3—C11	1.375 (3)	C20—C23	1.527 (4)
C1—C2	1.386 (4)	C20—C21	1.531 (4)
C1—H1A	0.9500	C20—C22	1.547 (4)
C2—C3	1.388 (4)	C21—H21A	0.9800
C2—H2A	0.9500	C21—H21B	0.9800
C3—C4	1.402 (4)	C21—H21C	0.9800
C3—C16	1.534 (4)	C22—H22A	0.9800
C4—C5	1.380 (4)	C22—H22B	0.9800
C4—H4A	0.9500	C22—H22C	0.9800
C5—C6	1.486 (3)	C23—H23A	0.9800
C6—C7	1.385 (4)	C23—H23B	0.9800
C7—C8	1.396 (4)	C23—H23C	0.9800
C7—H7A	0.9500	C24—C27	1.522 (4)
C8—C9	1.401 (4)	C24—C26	1.529 (4)
C8—C20	1.531 (4)	C24—C25	1.540 (4)
C9—C10	1.381 (4)	C25—H25A	0.9800
C9—H9A	0.9500	C25—H25B	0.9800
C10—C11	1.478 (4)	C25—H25C	0.9800
C11—C12	1.378 (4)	C26—H26A	0.9800
C12—C13	1.395 (4)	C26—H26B	0.9800
C12—H12A	0.9500	C26—H26C	0.9800
C13—C14	1.389 (4)	C27—H27A	0.9800
C13—C24	1.528 (4)	C27—H27B	0.9800
C14—C15	1.390 (4)	C27—H27C	0.9800
C14—H14A	0.9500	F1—B1	1.392 (4)
C15—H15A	0.9500	F2—B1	1.405 (4)
C16—C19	1.536 (4)	F3—B1	1.389 (3)
C16—C18	1.537 (4)	F4—B1	1.393 (3)
C16—C17	1.543 (4)		
			
N2—Pt1—N1	80.51 (9)	C16—C17—H17C	109.5
N2—Pt1—N3	81.26 (9)	H17A—C17—H17C	109.5
N1—Pt1—N3	161.70 (9)	H17B—C17—H17C	109.5
N2—Pt1—Cl1	179.44 (7)	C16—C18—H18A	109.5
N1—Pt1—Cl1	99.70 (6)	C16—C18—H18B	109.5
N3—Pt1—Cl1	98.51 (6)	H18A—C18—H18B	109.5
C1—N1—C5	118.6 (2)	C16—C18—H18C	109.5
C1—N1—Pt1	127.43 (19)	H18A—C18—H18C	109.5
C5—N1—Pt1	113.95 (17)	H18B—C18—H18C	109.5
C6—N2—C10	122.6 (2)	C16—C19—H19A	109.5
C6—N2—Pt1	119.14 (17)	C16—C19—H19B	109.5
C10—N2—Pt1	118.21 (17)	H19A—C19—H19B	109.5
C15—N3—C11	118.1 (2)	C16—C19—H19C	109.5
C15—N3—Pt1	128.79 (18)	H19A—C19—H19C	109.5
C11—N3—Pt1	112.93 (17)	H19B—C19—H19C	109.5
N1—C1—C2	121.7 (3)	C23—C20—C8	109.4 (2)
N1—C1—H1A	119.2	C23—C20—C21	108.7 (2)
C2—C1—H1A	119.2	C8—C20—C21	112.3 (2)
C1—C2—C3	121.0 (3)	C23—C20—C22	109.9 (3)
C1—C2—H2A	119.5	C8—C20—C22	108.7 (2)
C3—C2—H2A	119.5	C21—C20—C22	107.9 (2)
C2—C3—C4	116.7 (3)	C20—C21—H21A	109.5
C2—C3—C16	121.4 (2)	C20—C21—H21B	109.5
C4—C3—C16	121.8 (3)	H21A—C21—H21B	109.5
C5—C4—C3	120.6 (3)	C20—C21—H21C	109.5
C5—C4—H4A	119.7	H21A—C21—H21C	109.5
C3—C4—H4A	119.7	H21B—C21—H21C	109.5
N1—C5—C4	121.4 (2)	C20—C22—H22A	109.5
N1—C5—C6	114.2 (2)	C20—C22—H22B	109.5
C4—C5—C6	124.4 (2)	H22A—C22—H22B	109.5
N2—C6—C7	119.5 (2)	C20—C22—H22C	109.5
N2—C6—C5	112.1 (2)	H22A—C22—H22C	109.5
C7—C6—C5	128.4 (2)	H22B—C22—H22C	109.5
C6—C7—C8	119.9 (2)	C20—C23—H23A	109.5
C6—C7—H7A	120.0	C20—C23—H23B	109.5
C8—C7—H7A	120.0	H23A—C23—H23B	109.5
C7—C8—C9	118.6 (2)	C20—C23—H23C	109.5
C7—C8—C20	120.4 (2)	H23A—C23—H23C	109.5
C9—C8—C20	120.9 (2)	H23B—C23—H23C	109.5
C10—C9—C8	119.7 (2)	C27—C24—C13	111.9 (2)
C10—C9—H9A	120.2	C27—C24—C26	110.0 (3)
C8—C9—H9A	120.2	C13—C24—C26	109.9 (2)
N2—C10—C9	119.6 (2)	C27—C24—C25	108.4 (3)
N2—C10—C11	112.6 (2)	C13—C24—C25	107.5 (2)
C9—C10—C11	127.8 (2)	C26—C24—C25	109.0 (2)
N3—C11—C12	121.1 (2)	C24—C25—H25A	109.5
N3—C11—C10	114.8 (2)	C24—C25—H25B	109.5
C12—C11—C10	124.0 (2)	H25A—C25—H25B	109.5
C11—C12—C13	121.4 (2)	C24—C25—H25C	109.5
C11—C12—H12A	119.3	H25A—C25—H25C	109.5
C13—C12—H12A	119.3	H25B—C25—H25C	109.5
C14—C13—C12	116.4 (2)	C24—C26—H26A	109.5
C14—C13—C24	123.8 (2)	C24—C26—H26B	109.5
C12—C13—C24	119.7 (2)	H26A—C26—H26B	109.5
C13—C14—C15	120.7 (3)	C24—C26—H26C	109.5
C13—C14—H14A	119.6	H26A—C26—H26C	109.5
C15—C14—H14A	119.6	H26B—C26—H26C	109.5
N3—C15—C14	122.0 (2)	C24—C27—H27A	109.5
N3—C15—H15A	119.0	C24—C27—H27B	109.5
C14—C15—H15A	119.0	H27A—C27—H27B	109.5
C3—C16—C19	111.4 (2)	C24—C27—H27C	109.5
C3—C16—C18	111.8 (2)	H27A—C27—H27C	109.5
C19—C16—C18	108.9 (2)	H27B—C27—H27C	109.5
C3—C16—C17	107.7 (2)	F3—B1—F1	109.6 (2)
C19—C16—C17	108.2 (2)	F3—B1—F4	109.8 (2)
C18—C16—C17	108.7 (3)	F1—B1—F4	109.1 (2)
C16—C17—H17A	109.5	F3—B1—F2	109.4 (2)
C16—C17—H17B	109.5	F1—B1—F2	109.4 (2)
H17A—C17—H17B	109.5	F4—B1—F2	109.5 (2)

### Hydrogen-bond geometry (Å, °)

**Table:**

*D*—H···*A*	*D*—H	H···*A*	*D*···*A*	*D*—H···*A*
C1—H1A···F3^i^	0.95	2.51	3.330 (3)	145
C2—H2A···F1^i^	0.95	2.36	3.229 (3)	151
C7—H7A···F2^ii^	0.95	2.46	3.333 (3)	154
C17—H17B···F4^ii^	0.98	2.36	3.295 (3)	159
C27—H27C···F3^iii^	0.98	2.48	3.349 (4)	147
C9—H9A···F4	0.95	2.39	3.250 (3)	150
C12—H12A···F4	0.95	2.49	3.298 (3)	142

Symmetry codes: (i) −*x*+1, −*y*+1, −*z*+1; (ii) *x*−1/2, −*y*+1/2, *z*−1/2; (iii) *x*−1/2, −*y*+1/2, *z*+1/2.
